# Supplementary material for: Microglia: The Drunken Gardeners of Early Adversity
Source: Biomolecules. 2024 Aug 8;14(8):964. doi: 10.3390/biom14080964 (PMC11353196; doi:10.3390/biom14080964)
Supplement: Supplementary file 1 [file biomolecules-14-00964-s001.zip › biomolecules-3095253-supplementary.pdf]

Supplemental Information

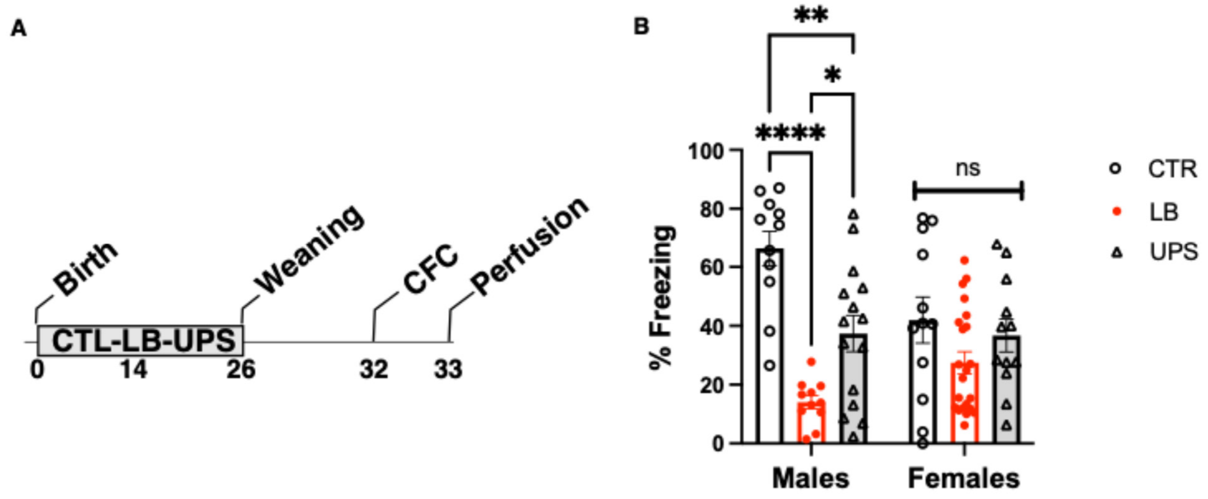

Figure S1. Mice exposed to LB show more severe deficits in contextual fear conditioning compared to UPS adolescent mice. (A) Mice were exposed to control (CTR), LB, or UPS from P0-25 as previously described [36]. At P32 mice were tested in the contextual fear conditioning [39]. (B) Contextual fear conditioning. A 3 x 2 ANOVA revealed significant Interaction between rearing and sex ($F (2, 77) = 5.75$, $P= 0.0047$). Tukey-HSD post-hoc analysis in males revealed significant reduction in freezing behavior in LB males compared to CTL males ($P <0.0001$) and UPS males ($P= 0.012$). No significant rearing effect was found in females. These findings are consistent with previous studies from our lab showing that adolescent LB males show more severe deficits in contextual fear conditioning compared to LB female littermates [39,60].
